# Social and Genetic Networks of HIV-1 Transmission in New York City

**DOI:** 10.1371/journal.ppat.1006000

**Published:** 2017-01-09

**Authors:** Joel O. Wertheim, Sergei L. Kosakovsky Pond, Lisa A. Forgione, Sanjay R. Mehta, Ben Murrell, Sharmila Shah, Davey M. Smith, Konrad Scheffler, Lucia V. Torian

**Affiliations:** 1 Department of Medicine, University of California San Diego, San Diego, California, United States of America; 2 New York City Department of Health and Mental Hygiene, New York, New York, United States of America; 3 Veterans Affairs Healthcare System San Diego, San Diego, California, United States of America; 4 Department of Mathematical Sciences, Stellenbosch University, Stellenbosch, South Africa; ETH Zürich, SWITZERLAND

## Abstract

**Background:**

Sexually transmitted infections spread across contact networks. Partner elicitation and notification are commonly used public health tools to identify, notify, and offer testing to persons linked in these contact networks. For HIV-1, a rapidly evolving pathogen with low per-contact transmission rates, viral genetic sequences are an additional source of data that can be used to infer or refine transmission networks.

**Methods and Findings:**

The New York City Department of Health and Mental Hygiene interviews individuals newly diagnosed with HIV and elicits names of sexual and injection drug using partners. By law, the Department of Health also receives HIV sequences when these individuals enter healthcare and their physicians order resistance testing. Our study used both HIV sequence and partner naming data from 1342 HIV-infected persons in New York City between 2006 and 2012 to infer and compare sexual/drug-use named partner and genetic transmission networks. Using these networks, we determined a range of genetic distance thresholds suitable for identifying potential transmission partners. In 48% of cases, named partners were infected with genetically closely related viruses, compatible with but not necessarily representing or implying, direct transmission. Partner pairs linked through the genetic similarity of their HIV sequences were also linked by naming in 53% of cases. Persons who reported high-risk heterosexual contact were more likely to name at least one partner with a genetically similar virus than those reporting their risk as injection drug use or men who have sex with men.

**Conclusions:**

We analyzed an unprecedentedly large and detailed partner tracing and HIV sequence dataset and determined an empirically justified range of genetic distance thresholds for identifying potential transmission partners. We conclude that genetic linkage provides more reliable evidence for identifying potential transmission partners than partner naming, highlighting the importance and complementarity of both epidemiological and molecular genetic surveillance for characterizing regional HIV-1 epidemics.

## Author Summary

Understanding the path over which viruses such as HIV have been transmitted may be crucial for directing public health resources and guiding policy decisions. Contact tracing of named sexual and injection drug-use partners of people recently diagnosed with HIV is an indispensible tool for reconstructing this transmission network. Viral genetic sequence data—routinely collected by public health agencies—can also be used to infer the dynamics of HIV transmission. We analyzed partner naming and viral genetic sequence data in 1342 people living with HIV in New York City reported to the New York City Department of Health and Mental Hygiene between 2006 and 2012. Genetically linked partners were more likely to be named partners than named partners were to be genetically linked. This finding indicates that genetic sequence data are better than partner naming data for reconstructing this viral transmission network. Importantly, the success rate in naming a genetically linked partner varied by transmission risk category (e.g., men who have sex with men, heterosexuals, and injection drug users). This study validates the use viral genetic sequences in reconstructing these viral transmission networks in a public health surveillance setting.

## Introduction

Analysis of pathogen genetics has shaped our understanding of the origin and spread of numerous infectious diseases, both viral and bacterial [1–5]. Genetic sequence analyses determined that the HIV-1 group M pandemic has zoonotic origins in chimpanzees [6, 7] and how HIV has migrated within and out of sub-Saharan Africa [8–10]. On a smaller scale, genetic sequence analyses are used to clarify dynamics of local HIV-1 transmission networks [11–16], which can be in turn used to target HIV prevention and intervention strategies [15, 17–19].

HIV transmission largely occurs along links in the social network connecting risk-sharing partners (e.g., injection drug users or sexual contacts), which represent potential routes of viral spread. The HIV transmission network is a subset of all of the risk exposure interactions. Techniques for reconstructing HIV transmission networks from viral sequence data provide an estimate of the unobserved transmission network [11, 15, 20–23] but cannot exclude unobserved intermediate or shared sources of infections [24] (i.e., two genetically linked individuals are close to each other in the true transmission network but not necessarily directly connected). Genetic distance based methods [11, 16, 21, 23] ascribe a putative transmission link to any pair of viral sequences that are within a predetermined genetic distance threshold, with the expectation that viral genetic diversity between transmission partners should approximate the diversity within the source partner [21], and allow for some degree of onward evolution in the recipient partner. Within a single person, HIV *pol* sequences (the genomic region routinely used in public health surveillance for antiretroviral drug resistance) tend not diverge more than 0.01 substitutions/site from the baseline sequence in the first 10 years of infection [25], and the total sequence divergence tends to be less than 0.02 substitutions/site [21]. Therefore, one would expect an epidemiologically meaningful genetic distance threshold for identifying transmission partners to fall between 0.01 and 0.02 substitutions/site. This range is consistent with previous observations [21, 26], but has not yet been empirically validated using named partners in a surveillance setting.

It is estimated that over 100,000 people residing in New York City, approximately 1.2% of the city’s population, are infected with HIV [27]. Among persons documented to be living with HIV/AIDS in New York City, the largest proportions comprise men who have sex with men (MSM, 37.4%) and Blacks/African Americans (44.4%). To identify new HIV cases and promote linkage to care, the Field Services Unit (FSU) of the New York City Department of Health and Mental Hygiene (DOHMH) interviews persons newly diagnosed with HIV infection (index cases), and elicits names of sexual or drug using partners in the past year (named partners). This partner tracing evinces only a fraction of all risk exposure connections that may have led to index HIV infections, or infections originating from index cases. After the interview, the named partners are notified of their exposure and offered HIV testing. When resistance testing is ordered by a physician with whom the index case or an HIV-positive partner has initiated care, the partial nucleotide sequence of the HIV *pol* gene is reported to DOHMH surveillance. These sequence data can then be used to reconstruct an HIV genetic transmission network [28, 29].

Key to designing and monitoring effective HIV prevention strategies is the identification of partners who transmitted HIV to each other; however, unambiguously identifying these partners is nearly impossible [24, 30, 31]. Our best indication that a transmission partner pair is correctly inferred is when the partners are linked both socially (named partners) and genetically (highly similar viral sequences). Previous investigations into inferred social and genetic networks in HIV focused on small, homogeneous populations of high-risk individuals [26, 32–35] and found that named partners were often not transmission partners; their viruses were too genetically dissimilar. Our study used an order of magnitude larger number of individuals (1342 people), with diverse risk factors, to investigate the use of a genetic distance threshold for identifying potential transmission partners (i.e., partners with a direct or indirect epidemiological connection) in a surveillance setting. We validate a range of biologically and epidemiologically plausible genetic distance thresholds and find that the degree of concordance between social (i.e., named partner) and genetic networks in New York City is relatively low and varies by risk factor and race/ethnicity. Based on these results, we suggest avenues to improve HIV surveillance and public health intervention activities.

## Methods

### Index case and named partner population

The FSU interviewed index cases diagnosed with HIV in the previous three months and elicited the names of partners who had engaged in sexual activity or injection drug use (IDU) with the index case in the previous 12 months. Named partners were contacted and referred to care; many of these named partners were also index cases in this population. Only the primary transmission risk factor was considered when classifying index cases and named partners whose genotype was reported to surveillance. For classification purposes, history of injection drug use took precedence over sexual risk behavior. Persons who did not report high-risk sexual activity were classified as having an unknown risk factor. Disease stage at diagnosis was assigned using BED testing, which is capable of providing a reasonable picture of HIV population-level incidence in the United States [36, 37]. HIV-1 subtyping was performed using SCUEAL [38]. For the purpose of this analysis, HIV-1 sequences were classified into B or non-B subtypes. Multivariate and univariate logistic regression analysis was used to model the probability of an index case being genetically linked to at least one named partner.

### Network analysis

To construct the genetic transmission network, we used HIV-TRACE (www.hivtrace.org), following a procedure described previously [23]. First, all HIV sequences were aligned to the HXB2 (GenBank accession K03455) reference sequence (coordinates: 2253–3869) using an extension of the Smith-Waterman algorithm [39], which aligns nucleotide sequences by considering amino-acid translations of constituent codons and corrects for possible frameshifts and sequencing errors; as insertions and deletions are rare in this region and phylogenetically uninformative, we filtered them from downstream analyses. The evolutionary conservation of length in this genomic region permits pairwise alignment as a timesaving measure.

Next, we calculated the pairwise Tamura-Nei 93 (TN93; [40]) genetic distances among all sequences. TN93 genetic distance was used because it can be computed rapidly via a closed-form solution (i.e., not involving a numerical optimization) that requires only counts of aligned nucleotide pairs as inputs and is the most complex evolutionary model (i.e., two types of transitions rates, a transversion rate, and unequal base frequencies) that admits such a closed form solution. Furthermore, for distances ≤0.05 substitutions/site, all commonly used nucleotide substitution models produce nearly identical estimates [41]. We then placed an edge (link) connecting pairs of sequences that fell below a distance threshold. Connected components of the resultant transmission network were interpreted as individual transmission clusters. The potential confounding effect of convergent evolution for drug resistance was assessed by repeating the analysis after excluding 48 codon positions in *protease* and *reverse transcriptase* associated with drug resistance [42].

When calculating genetic distance between sequences, we resolved all IUPAC defined nucleotide ambiguities (i.e., non-ACGT) to the corresponding nucleotide in the other sequences (i.e., Y is zero distance from both C and T). Following the protocols established by the Los Alamos National Laboratory HIV Sequence Database [http://www.hiv.lanl.gov/] to curate problematic sequences, we excluded from the study 13 persons whose viral sequences contained ≥5% ambiguities.

To investigate the distribution of genetic distances among named partners, a mixture distribution was defined as the weighted sum of a Gamma distribution (mean μ parameter and standard deviation σ), and a Gaussian (normal) distribution (with mean μ and standard deviation σ), and the parameter p controlling the mixture weight (S1 Table). The Metropolis-Hastings algorithm was used to estimate the parameters of this distribution. To improve mixing, the Gamma parameters were transformed into means and standard deviations, and a uniform (improper) prior was used over all parameters in this transformed parameter space. 200,000 MCMC samples were drawn, and the first 10,000 were discarded as burn-in. Mixing was assessed visually.

### Ethics statement

This study was a routine analysis of surveillance, laboratory, and partner services data reported to the Department of Health as mandated by New York State Public Health Law. All patient and partner matching was performed by authorized surveillance personnel. Cases and partners were assigned identification numbers that were unique to this analysis and could only be linked back to the original data by the same authorized personnel, in essence de-identifying the analytic dataset. Consent was not required because these data were collected and analyzed in the course of routine public health surveillance. The Institutional Review Board (IRB) of the University of California, San Diego Human Research Protections Program reviewed this study and certified it exempt from IRB review, stating that this research involved “the study of existing data…and the information was provided in such a way that the subjects cannot not be identified, directly or through identifiers linked to the subjects.”

## Results

### Study population and Field Services Unit (FSU) partner tracing

Between 2006 and 2012, the FSU identified 756 index cases who named 586 unique HIV-positive partners who also had an HIV-1 *pol* sequence reported to the DOHMH. This study population was comprised mostly of individuals reporting MSM risk factor (44%), then heterosexual risk (32%) and injection drug use (8%). MSM index cases named more partners on average (Table 1) and were slightly more likely than heterosexual females to have named partners for whom an HIV genotype was available (incidence rate ratio = 1.18; 95% confidence interval: 1.00–1.39; Poisson regression; *p* = 0.05). The mean number of named partners who were genotyped did not vary significantly by race/ethnicity.

**Table 1:** Mean number of named and genotyped partners by index case demographic characteristics.

Demographic	Category^1^	Index cases	Mean number of named partners (range)	Mean number of genotyped partners (range)
Total	-	756	2.3 (1–62)	1.1 (1–12)
Risk	Hetero (F)	215	1.6 (1–8)	1.0 (1–2)
	Hetero (M)	92	1.5 (1–6)	1.1 (1–2)
	MSM	339	2.9 (1–50)	1.2 (1–5)
	IDU (F)	15	1.6 (1–4)	1.0 (1–1)
	IDU (M)	35	2.0 (1–9)	1.0 (1–2)
	Other/Unknown	60	2.6 (1–62)	1.3 (1–12)
Race	Black	390	2.1 (1–21)	1.1 (1–4)
	Hispanic	306	2.3 (1–62)	1.1 (1–12)
	White/Other	60	3.3 (1–50)	1.1 (1–2)

Hetero, heterosexual; MSM, men who have sex with men; IDU, injecting drug user

^1^Demographic categories reflect index case

### Genetic distance threshold

**Fig 1:**
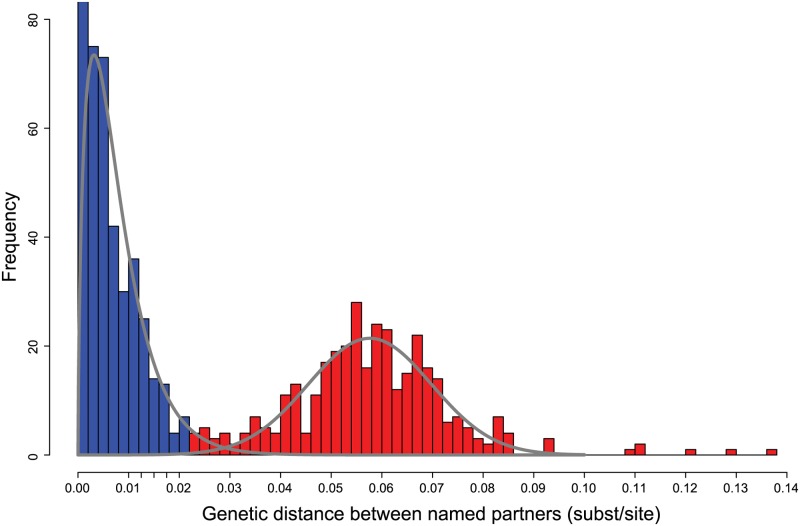
Genetic distance (Tamura-Nei 93; TN93) separating index cases and named partners. Gray lines show the best-fitting mixture distribution. Additional tick marks indicating epidemiologically plausible thresholds between 0.01 and 0.02 substitutions/site are shown on x-axis. Blue denotes potential transmission partners (≤0.02 substitutions/site). Red denotes partners with “random” within or between subtype viral divergence.

The distribution of genetic distances between viral sequences from index cases to all of their named partners was bimodal (Fig 1), with the left component representing potential transmission partners and the right component representing sequences no more similar to each other than two random isolates of the same subtype (i.e., 0.03 and 0.08 substitutions/site). We fit a mixture distribution, the weighted sum of Gamma and Gaussian (normal) distributions, to objectively assign distances to categories. We found that 99.9% of the probability mass of the fitted normal component (mean = 0.058 substitutions/site; standard deviation = 0.012 substitutions/site), representing unrelated pairings, was >0.02 substitutions/site. The Gamma distribution, representing potential transmission partners, had a mean 0.008 substitutions/site and a standard deviation of 0.006 substitutions/site (see S1 Table for more detail).

**Fig 2:**
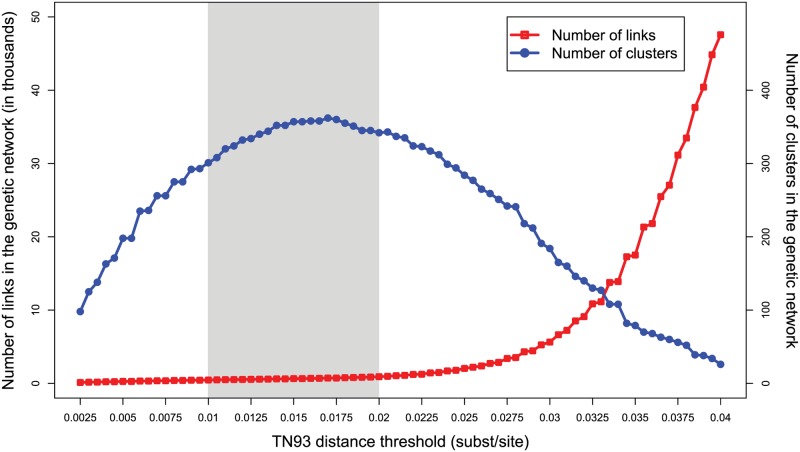
Number of genetic links and transmission clusters, as a function of the TN93 distance threshold. The epidemiologically plausible range of thresholds between 0.01 and 0.02 substitutions/site is highlighted in gray.

We performed our initial analyses using a genetic distance threshold of 0.0175 substitutions/site, because this distance identifies the maximum number of clusters in the genetic network (Fig 2). Above 0.0175 substitutions/site, clusters begin to coalesce and the network loses resolution. Nonetheless, we also explored the effect of using more conservative and liberal distance thresholds ranging between 0.01 and 0.02 substitutions/site.

### Drug resistance associated mutations

**Fig 3:**
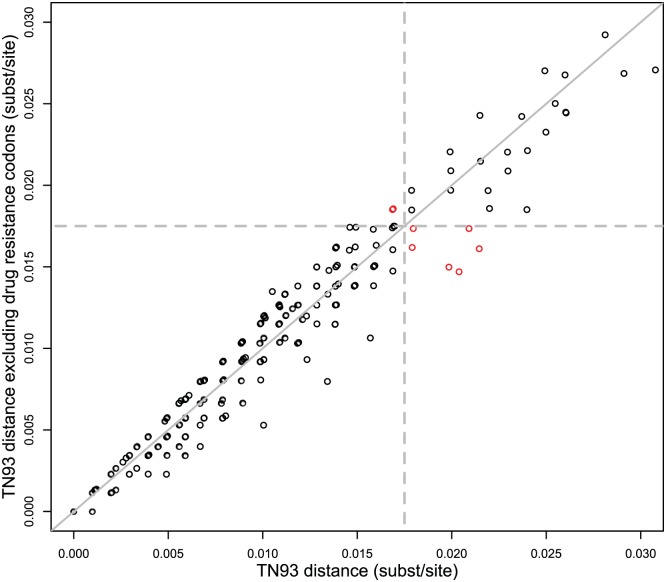
TN93 genetic distances between named partners ≤0.03 substitutions/site including and excluding codons associated with drug resistance. Disagreement in classification (linked/unlinked) between distance models is shown in red. The line x = y is shown in solid gray. Dashed lines denote 1.75% genetic distance threshold.

Determination of genetic linkage was robust to the inclusion or exclusion of sites associated with drug resistance (Fig 3), a possible confounding factor due to convergent evolution for mutations conferring drug resistance. The agreement in classification (i.e., linked or not linked) of partner pairs whose genetic distance was below the lower threshold for random within subtype B variation (i.e., 0.03 substitutions/site) was 98% when their genetic distance was calculated with or without codons associated with drug resistance using the 0.0175 distance threshold. If we were to exclude codons associated with drug resistance in the distance calculation, two partner pairs would become unlinked and an additional six pairs would become linked. All eight of these potential transmission partners that changed linkage due to inclusion or exclusion of codons associated with drug resistance had distances near the cutoff threshold. This pattern of general agreement in inference of partner pairs with or without the inclusion of codons associated with drug resistance held across a range of distance thresholds between 0.01 and 0.02 substitutions/site (Table 2).

**Table 2:** Sensitivity to genetic distance thresholds ranging between 0.01 and 0.02 substitutions/site.

Outcome	Genetic distance threshold (substitutions/site) using TN93 distance				
	0.01	0.0125	0.015	0.0175	0.02
Genetic linkage agreement when excluding codons associated with drug resistance	95%	96%	96%	98%	98%
Index cases genetically linked to ≥1 named partner (%)	46%	53%	57%	59%	60%
Index cases genetically linked to reciprocally named partner (%)	65%	72%	76%	79%	79%
Genetic links supported by partner namings (%)	65%	62%	57%	53%	43%
Partner namings supported by genetic links (%)	37%	42%	46%	48%	49%

### Comparison of named partner and genetic networks

**Fig 4:**
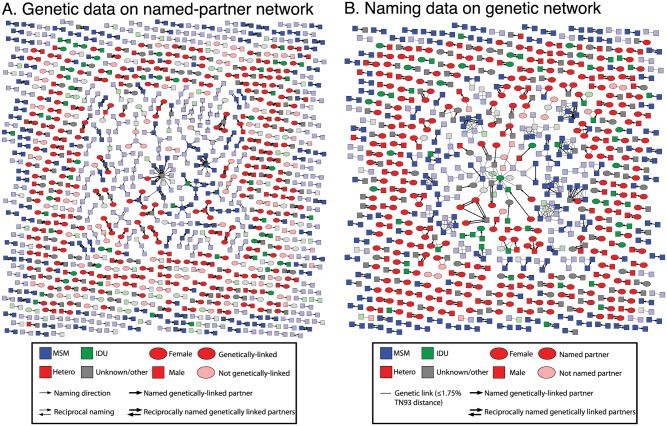
Concordance between named partner and genetic networks. (A) Genetic data mapped onto named partner network. Edges indicate partner naming. (B) Partner naming data mapped onto genetic network. Edges indicate genetic linkage (≤0.0175 substitutions/site).

We constructed two networks in which nodes represent index cases and partners: a named partner network and a genetic linkage network (Fig 4). To investigate which named partnerships are compatible with direct transmission, we mapped the genetic data to the named partner network (Fig 4A).

Of the 651 recorded partner namings (i.e., edges in the named partner network), the genetic data provide corroborating evidence for transmission along 48% (310/651) of these edges; the genetic data effectively rule out transmission along the remaining 52% (341/651) of edges. Importantly, these genetic distances that rule out transmission were not just slightly above the threshold, but overwhelmingly represented random within subtype-diversity (0.03–0.08 substitutions/site divergence; Fig 1). To investigate which genetic links were supported by partner tracing, and therefore more likely to represent direct transmission events, we also mapped the partner naming data to the genetic network (Fig 4B). In the genetic network, we found 736 edges: pairs of viral sequences that were ≤0.0175 substitutions/site apart. Partner naming provided evidence for direct transmission along 53% (388/736) of edges in this genetic network. It is important to recognize that lack of direct partner naming does not definitively rule out direct transmission but could be attributed to incomplete partner naming or other sampling deficiencies. We also mapped the social and genetic data onto a single network (S1 Fig) to provide another perspective on the overlap and complementarity between these networks.

**Fig 5:**
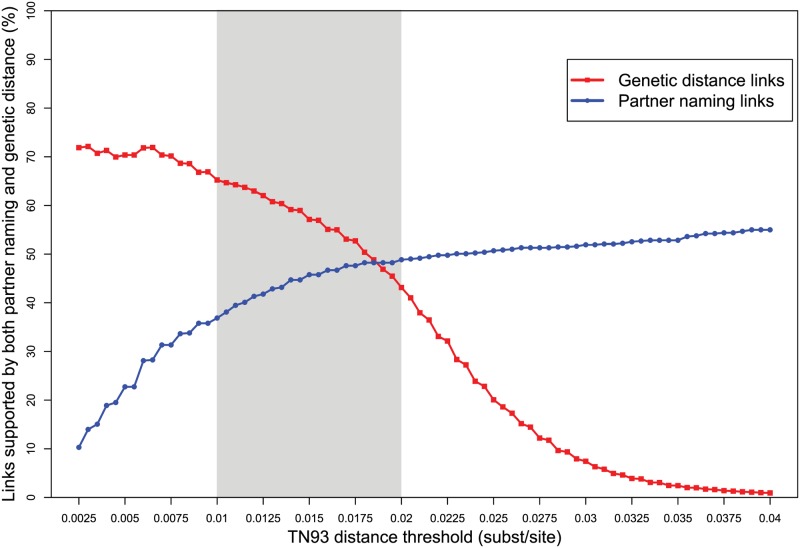
Proportion of partner namings and genetic links that agree, in relation to the TN93 genetic distance threshold. The epidemiologically plausible range of thresholds between 0.01 and 0.02 substitutions/site is highlighted in gray.

As the genetic distance threshold became more stringent (e.g., 0.01 substitutions/site), there were fewer genetic links (466 edges), and a greater proportion of them were supported by partner naming: 65% (304/466) of links (Fig 5; Table 2). At this conservative threshold, only 37% (240/651) of partner namings corresponded to a genetic link. Using a more liberal but still epidemiologically plausible distance threshold of 0.02 substitutions/site, a more densely connected network with 918 genetic links was produced (Fig 2). As a consequence, a smaller proportion of these genetic links were supported by partner naming (43%; 396/918), and more partner namings were supported by genetic evidence (50%; 324/651). At the genetic distance threshold commonly used for investigating HIV transmission network dynamics in a surveillance population (i.e., 0.015 substitutions/site) [16, 43, 44], the results were similar (Table 2; see S2 Fig for a detailed comparison at 0.015 substitutions/site). Across the entire range of validated genetic distance thresholds for establishing potential transmission partners, the genetic evidence indicates that partner naming did not identify potential transmission partners in more than half the cases (Fig 5; Table 2).

As the genetic distance threshold increases, connections in the genetic network become less informative. Additional edges, rather than forming new clusters, tend to fill in already existing clusters and bridge distinct clusters, creating accreted dense clusters in which nearly all members are directly linked to each other, an epidemiologically uninformative scenario. This unwanted scenario occurred above the epidemiologically plausible range of 0.01 to 0.02 substitutions/site, thus providing additional evidence that a threshold within this range is ideal for identifying potential transmission partners and clusters. Importantly, even when the genetic distance threshold was extremely permissive (e.g., 0.04) and the number of genetic links increased dramatically (47,573 edges; Fig 2), the proportion of partner namings supported by genetic links was only slightly more than half: 55% (358/651) (Fig 5). In other words, nearly half of named partners are infected with a virus that is no more related to the index case than a random isolate of the same subtype.

### Correlates of genetic linkage to named partner

Of the 756 index cases, 449 (59%) were genetically linked to at least one named partner at 0.0175 substitutions/site. The frequency at which an index case was genetically linked to one or more named partners varied by risk group (Table 3). Heterosexual female and male index cases, were the most likely to be genetically linked to at least one named partner (77% of index cases). In contrast, MSM index cases were significantly less likely than heterosexual females to be genetically linked to at least one named partner (42% of index cases; *p* < 0.001). Index cases who reported injection drug use were also less likely to be genetically linked to named partners (53% of females and 43% of males; *p* = 0.061 and *p* < 0.001, respectively). Although Black/African American index cases were significantly less likely to be genetically linked to a named partner than Hispanics or Whites/Other (*p* < 0.001 and *p* = 0.014), the magnitudes of the differences by race/ethnicity were smaller than those among risk groups (Table 3). Further, the difference between risk groups was not driven solely by race/ethnicity. When the logistic regression was restricted to Black/African American index cases or excluded Black/African American index cases the adjusted odds ratios were essentially unchanged: 0.254 (0.143–0.451) versus 0.214 (0.115–0.398).

**Table 3:** Logistic regression analysis of index case being genetically-linked to at least one of their named partners.

Demographic	Category^1^	Index cases	Genetically linked to ≥1 named partner, n (%)	Not genetically linked to a named partner, n (%)	Odds Ratio^2^	95% confidence interval	*p*-value
Total	-	756	449 (59%)	307 (41%)	-	-	-
Risk	Hetero (F)	215	166 (77%)	49 (23%)	1	-	-
	Hetero (M)	92	71 (77%)	21 (23%)	1.06	0.58–1.94	0.861
	MSM	339	143 (42%)	196 (58%)	0.22	0.14–0.33	<0.001
	IDU (F)	15	8 (53%)	7 (47%)	0.35	0.12–1.05	0.061
	IDU (M)	35	15 (43%)	20 (23%)	0.20	0.09–0.44	<0.001
	Other/Unknown	60	46 (77%)	14 (23%)	1.16	0.57–2.34	0.685
Race	Black	390	209 (54%)	181 (46%)	1	-	-
	Hispanic	306	204 (67%)	102 (33%)	1.95	1.35–2.83	<0.001
	White/Other	60	36 (60%)	24 (40%)	2.19	1.17–4.08	0.014
Country of birth	USA	489	276 (56%)	213 (44%)	1	-	-
	Foreign	211	138 (65%)	73 (35%)	1.18	0.80–1.74	0.399
	US dependency	54	34 (63%)	20 (37%)	0.78	0.39–1.55	0.477
	Unknown	2	1 (50%)	1 (50%)	0.43	0.02–9.09	0.589
Subtype	B	700	413 (59%)	287 (41%)	1	-	-
	Non-B	56	36 (64%)	20 (36%)	0.93	0.50–1.74	0.831
Stage at diagnosis	Chronic	207	119 (57%)	88 (43%)	1	-	-
	Acute/early	126	90 (71%)	36 (29%)	1.74	1.02–2.97	0.040
	Unknown	423	240 (57%)	183 (43%)	1.05	0.72–1.53	0.802
AIDS status in 2013	Non-AIDS	432	258 (60%)	174 (40%)	1	-	-
	AIDS	324	191 (59%)	133 (41%)	0.85	0.60–1.19	0.338
Age at diagnosis	-	-	-	-	1.00	0.99–1.02	0.418
Named partners	-	-	-	-	0.91	0.85–0.99	0.023
Genotyped partners	-	-	-	-	1.34	0.90–2.01	0.154

Hetero, heterosexual; MSM, men who have sex with men; IDU, injecting drug user

^1^Demographic categories reflect index case

^2^Model adjusted odds ratio

There was an increased rate of genetic linkage to a named partner when the index case had been diagnosed during the acute or early stages of infection (*p* = 0.040). It is unclear whether this difference was due to increased infectiousness or transmission risk during acute/early infection [45], better ability to recall recent high-risk behavior and partners, or limited sequence evolution since transmission [46]. No significant association was found between genetic linkage to a named partner and country of birth, HIV-1 subtype, AIDS status as of 2013, and age at diagnosis.

The associations between genetic linkage to at least one named partner were generally consistent between the multivariate regression analysis (described above) and the univariate models (S2 Table). Foreign-born individuals were slightly more likely to be genetically linked to at least named partner in the univariate analysis, but this association is not found in the multivariate analysis. No differences in the importance of risk factor, race/ethnicity, stage of diagnoses, subtype, or AIDS status were found between the models. These results were also qualitatively similar when we compared the percentages of genetically linked named partners for each index case (S3 Table), which accounts for different mean numbers of named partners among demographic groups.

### Genetic distance by risk factor

**Fig 6:**
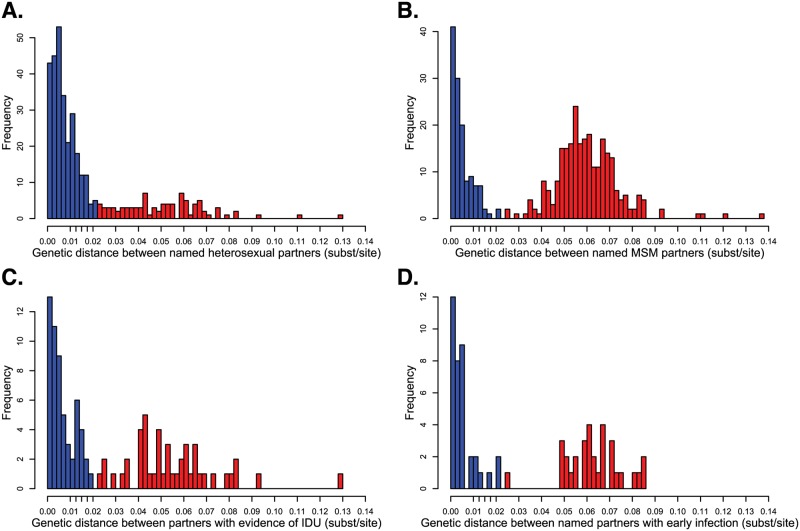
Genetic distance (TN93) separating index cases and named partners in select risk groups. (A) Genetic distance between named heterosexual partners. (B) Genetic distance between named MSM partners. (C) Genetic distance between named partners where at least one partner reported injection drug use (IDU). (D) Genetic distance between named partners where at least one partner was diagnosed with acute or early HIV infection. Additional tick marks on the x-axis indicate epidemiologically plausible thresholds between 0.01 and 0.02 substitutions/site are shown on x-axis. Blue denotes potential transmission partners (≤0.02 substitutions/site). Red denotes partners with “random” within or between subtype viral divergence.

As illustrated earlier (Fig 1), the genetic distance between viruses from index cases and named partners can be distinguished by two prominent modes: potential transmission partners and random within subtype variants. This bimodal distribution was less evident when this analysis was restricted to heterosexual couples with no evidence of IDU (Fig 6A). This difference may be partly attributable to the relatively high proportion of heterosexual males and females that named a partner with a closely related virus (77%; Table 3). In MSM and partners who reported IDU, there is a clear grouping of index cases who named partners whose viruses were no more genetically similar than random isolates of the same subtype (Fig 6B and 6C). Among partner pairs where at least one member was diagnosed with acute or early HIV infection, we also we saw a marked distinction between index cases who named potential transmission partners and index cases who identified partners with distantly related isolates (Fig 6D). Like heterosexual index cases, an index case with acute/early HIV diagnosis was highly predictive of naming at least one genetically linked partner (Table 3). However, unlike in heterosexual partners, the genetic distance separating partners with at least one acute/early diagnosis had a clear bimodal distribution. This observation suggests that ability to identify potential transmission partners is not strongly dependent on acute/early diagnosis.

### Sensitivity analysis

Across the range of epidemiologically plausible distance thresholds (i.e., 0.01 to 0.02 substitutions/site), the proportion of index cases who were genetically linked to at least one named partner remained fairly consistent (46–60%; Table 2). More importantly, the statistical associations found between genetic linkage and risk factor, race/ethnicity, and stage of infection were qualitatively similar.

Index cases who named more partners were slightly less likely to be genetically linked to any of these partners (*p* = 0.023; Table 3). There was no significant relationship between an index case being genetically linked to at least one named partner and the number of named partners who were genotyped. Because inclusion in our study required at least one named partner to have a reported genotype, it could be possible that MSM index cases had a lower probability of genetic linkage simply because a lower proportion of their named partners had a reported genotype, compared to other risk groups. Therefore, we reanalyzed the data restricting the analysis to index cases who provided only one named partner. MSM index cases who named only one partner were still less likely to be genetically linked to their single named partner than heterosexual index cases (*p* < 0.001; S4 Table).

### Reciprocally named partners

A total of 239 HIV-positive named partners were interviewed by the FSU to elicit additional named partners; these persons were also considered index cases in their own right. For 189 index cases, their named partner independently named the original index case during the interview. Such reciprocal naming increased the odds that named partners would be genetically linked compared to unidirectionally named partners: model adjusted odds ratio = 3.72 (95% confidence interval: 2.43–5.69). Nearly 4 out of 5 (149/189) reciprocally named partner pairs were also genetically linked (Table 4). The rate of genetic linkage increased for all risk groups relative to unidirectional naming: 90% of the time for female heterosexual index cases and 64% of the time for MSM index cases. This pattern was observed across a range of genetic distance thresholds (Table 2).

**Table 4:** Index cases who were reciprocally named by their named partner (n = 189 partner pairs).

Demographic	Category^1^	Index cases	Genetically linked, n (%)	Not genetically linked, n (%)
Total	-	189	149 (79%)	40 (21%)
Risk	Hetero (F)	51	46 (90%)	5 (10%)
	Hetero (M)	28	24 (86%)	4 (14%)
	MSM	78	50 (64%)	28 (36%)
	IDU (F)	6	5 (83%)	1 (17%)
	IDU (M)	4	3 (75%)	1 (25%)
	Other/Unknown	22	21 (95%)	1 (5%)
Race^3^	Black	84	57 (68%)	27 (32%)
	Hispanic	92	81 (88%)	11 (12%)
	White/Other	13	11 (85%)	2 (15%)

Hetero, heterosexual; MSM, men who have sex with men; IDU, injecting drug user

^1^Demographic categories are tabulated based on index cases

## Discussion

We analyzed an unprecedentedly expansive and detailed partner tracing and HIV sequence dataset collected from 1342 HIV-infected persons in New York City between 2006 and 2012 and determined an empirically justified and epidemiological plausible range of genetic distance thresholds for identifying potential transmission partners. Over this range of genetic distance thresholds, if an index case named an identifiable partner who tested positive for HIV, a genetic link indicating transmission was absent more than 50% of the time. Therefore, according to the genetic data, at least half of named partners are not plausible transmission partners; their HIV-1 sequences are no more similar to the HIV-1 sequence isolated from the corresponding index case sequences than to a randomly chosen sequence of the same HIV-1 subtype. Encouragingly, if both partners named each other, the odds of genetic linkage increased significantly across all transmission risk factors.

We found that despite naming more partners per index case than heterosexuals, MSM were less likely to name any partners with genetically linked viruses. Black/African American index cases, compared with Hispanic and White/Other index cases, were less likely to name a partner with genetically linked viruses. When an index case was reciprocally named by their named partner, the odds of infection with a genetically linked virus increased for all risk and race/ethnicity groups. This observation highlights the importance of reciprocal naming in identifying potential transmission partners.

Although one should never expect perfect concordance between social and genetic networks, their relative overlap provides insight into their respective usefulness in guiding public health interventions. Genetic transmission links were supported between 43% and 65% of the time by partner tracing information, depending on the genetic distance threshold. Importantly, lack of naming does not contradict the genetic inference. Rather, it can be attributable to an absence of evidence, not evidence of absence. If two genetically linked individuals are not named partners, it may be due to incomplete partner enumeration, thus highlighting the difficulty in eliciting the names of transmission partners. The proportion of genetic links supported by partner naming data can be viewed as the lower bound on the proportion of genetic links that represent recent transmission events in the network. Therefore, over a range of epidemiologically plausible genetic distance thresholds, genetic data are at least as good, and almost certainly better, than partner tracing data for inferring HIV recent transmission partners, despite known issues in genetic sequence analysis (e.g., spurious transitive edges in densely connected clusters). Furthermore, genetic data can help filter out reported at-risk contacts that did not lead to transmission, providing independent evidence for absence of a transmission event between named partners.

We recommend using a genetic distance threshold between 0.01 and 0.02 substitutions/site for identifying potential transmission partners in a surveillance setting. Admittedly, the approach to validating this cutoff will be biased towards detecting more recent transmission partners; however, this bias can be advantageous from a public health perspective, where the goal may be to identify recent partners in a growing transmission cluster. It is nontrivial to define a specific threshold systematically, because threshold tuning is always an exercise in balancing sensitivity and specificity. For example, when using genetic distance comparisons in typical HIV surveillance datasets that are not restricted to named partners, we suggest using a more conservative genetic threshold (e.g., 0.01 to 0.015) to identify partners with an epidemiologically meaningful relationship. Nevertheless, our findings confirm previous work showing that genetic distance information can be used to identify potential transmission partners in both early [17, 26] and chronic [11, 15] infection. These findings also suggest that using a genetic distance threshold without relying on phylogenetic tree inference and interpretation (as in [16, 21, 23, 43, 44]) is a valid approach for identifying potential transmission partners.

Because financial and personnel resources needed for exhaustive partner tracing are not feasible, genetic transmission networks inferred from sequences collected during routine drug resistance screening represent a relatively easy and inexpensive method for reconstructing the transmission history of HIV. Nevertheless, our study shows that there is great value in collecting partner-tracing data. For example, many named partners who, according to genetic data collected after diagnosis, are unlikely to be direct transmission partners, are HIV-positive but undiagnosed/unaware until they are offered HIV testing through the partner services program offered by the FSU. Therefore, partner tracing discovers HIV-infected persons who may not have been previously known to public health officials, allowing these persons to enter care and expanding the scope of potential intervention across the transmission network. Identifying these persons, regardless of whether they were previously known to surveillance, allows field workers to contact them, ensure linkage or return to care, elicit and notify partners, and expand the network of persons in the city that are receiving public health services. For example, 15.3% (81/448) of named partners in the genetic network linked only to someone other than the index case who named them; therefore, it is possible that simply being a named partner indicates an elevated risk status and possibly increased importance in the network (i.e., an intervention-worthy case) [26, 33].

To maximize the probability of finding transmission partners, the geographic scope of surveillance and comparison should be as broad as possible. In other studies of HIV transmission in citywide epidemics in Uganda, United Kingdom, and the United States [17, 47–49], only 30% of new infections can be identified as having originated in a given city. Even though the FSU interviews partners in the greater metropolitan region surrounding New York City, it is likely that many transmission partners reside outside their reach. Expanded collaboration and coordination among public health departments could help identify these geographically dispersed transmission events.

In addition, index cases who are not genetically linked to any of their named partners may be attractive candidates for additional interviews to identify potential transmission partners and expand network surveillance. On the other hand, because persons who named more partners were less likely to be genetically linked to any of their named partners, additional follow-up interviews may fail to meaningfully expand the scope of the network. To determine the value of repeated interviews, it will be important to record whether their “second round” of named partners were HIV-positive, linked to care, and yielded a viral genotype.

Identifying transmission partners is of critical importance in combating HIV, because HIV transmission networks tend to be best described by preferential attachment (or more generally, scale-free) models [15, 23], in which certain groups of highly connected individuals are central to the propagation of the epidemic. This scale-free property is inherited from the underlying social and contact networks [50]. Importantly, scale-free networks cannot be reliably disrupted by incomplete random interventions: only network-guided interventions targeted at transmission hubs (i.e., core transmitters and associated venues) can successfully combat the epidemic [15, 17, 50]. By identifying clusters that grew disproportionately in the past, we may be able to predict which clusters hold the greatest potential for future growth. Unfortunately, the time delay between HIV diagnosis/interview and genotype acquisition by the DOHMH (i.e., 6 months in our study, though this delay has shrunk since 2012) represents an impediment to prompt network-based intervention. Point-of-diagnosis-genotyping coupled with real-time genetic network analysis to identify potential transmission partners could help interdict ongoing transmission and target prevention, linkage to care, and treatment more effectively (as recent suggested in [18]).

## Supporting Information

S1 Table Parameter estimates for the mixture distribution of Gamma and Gaussian (normal) distributions. (DOCX)

S2 Table Univariate logistic regression analysis of index case being genetically-linked to at least one of their named partners. (DOCX)

S3 Table Multivariate regression analysis of index cases being genetically-linked to their named partners. (DOCX)

S4 Table Logistic regression analysis of index case being genetically-linked to at least one of their named partners for index cases who named only 1 partner. (DOCX)

S1 Fig Combined named partner and genetic (≤0.0175 substitutions/site) networks. Shaded nodes are genetically linked to at least one named partner. Bold edges indicate partner naming that is supported by genetic distance. Edges with arrows indicate direction of partner naming. Edges without arrows are links supported only by genetic distance.
(EPS)

S2 Fig Concordance between named partner and genetic (≤0.015 substitutions/site) networks. (A) Genetic data mapped onto named partner network. Edges indicate partner naming. (B) Partner naming data mapped onto genetic network. Edges indicate genetic linkage.
(EPS)
