# Supplementary material for: Social and Genetic Networks of HIV-1 Transmission in New York City
Source: PLoS Pathog. 2017 Jan 9;13(1):e1006000. doi: 10.1371/journal.ppat.1006000 (PMC5221827; doi:10.1371/journal.ppat.1006000)
Supplement: S1 Table — (DOCX) [file ppat.1006000.s001.docx]

**Table S1. Parameter estimates for the mixture distribution^1^ of Gamma and Gaussian (normal) distributions.**

| **Parameter** | **Abbreviation** | **Posterior mean** | **Lower 95% credible interval** | **Upper 95% credible interval** |
| --- | --- | --- | --- | --- |
| Mixture weight | p | 0.550 | 0.511 | 0.588 |
| Gamma mean | μ | 0.008 | 1.425 | 1.895 |
| Gamma standard deviation | σ | 0.006 | 0.004 | 0.006 |
| Gaussian mean | μ’ | 0.058 | 0.056 | 0.059 |
| Gaussian standard deviation | σ’ | 0.012 | 0.011 | 0.013 |

^1^mixture(p,μ,σ,μ’,σ’) = p $*$ Gamma(μ,σ) + (1 – p) $*$ Gaussian(μ’,σ’)
