# Supplementary material for: Social and Genetic Networks of HIV-1 Transmission in New York City
Source: PLoS Pathog. 2017 Jan 9;13(1):e1006000. doi: 10.1371/journal.ppat.1006000 (PMC5221827; doi:10.1371/journal.ppat.1006000)
Supplement: S2 Table — (DOCX) [file ppat.1006000.s002.docx]

**Table S2. Univariate logistic regression analysis of index case being genetically-linked to at least one of their named partners.**

| **Demographic** | **Category^1^** | **Index cases** | **Genetically linked to ≥1 named partner, n (%)** | **Not genetically linked to a named partner, n (%)** | **Odds Ratio^2^** | **95% confidence interval** | ***p*-value** |
| --- | --- | --- | --- | --- | --- | --- | --- |
| Total | - | 756 | 449 (59%) | 307 (41%) | - | - | - |
| Risk | Hetero (F) | 215 | 166 (77%) | 49 (23%) | 1 | - | - |
|  | Hetero (M) | 92 | 71 (77%) | 21 (23%) | 1.00 | 0.56–1.79 | 0.995 |
|  | MSM | 339 | 143 (42%) | 196 (58%) | 0.22 | 0.15–0.32 | <0.001 |
|  | IDU (F) | 15 | 8 (53%) | 7 (47%) | 0.34 | 0.12–0.98 | 0.045 |
|  | IDU (M) | 35 | 15 (43%) | 20 (23%) | 0.22 | 0.11–0.46 | <0.001 |
|  | Other/Unknown | 60 | 46 (77%) | 14 (23%) | 0.97 | 0.49–1.91 | 0.930 |
| Race | Black | 390 | 209 (54%) | 181 (46%) | 1 | - | - |
|  | Hispanic | 306 | 204 (67%) | 102 (33%) | 1.73 | 1.27–2.36 | 0.001 |
|  | White/Other | 60 | 36 (60%) | 24 (40%) | 1.30 | 0.75–2.26 | 0.354 |
| Country of birth | USA | 489 | 276 (56%) | 213 (44%) | 1 | - | - |
|  | Foreign | 211 | 138 (65%) | 73 (35%) | 1.46 | 1.04–2.04 | 0.027 |
|  | US dependency | 54 | 34 (63%) | 20 (37%) | 1.31 | 0.73–2.34 | 0.359 |
|  | Unknown | 2 | 1 (50%) | 1 (50%) | 0.77 | 0.48–12.41 | 0.855 |
| Subtype | B | 700 | 413 (59%) | 287 (41%) | 1 | - | - |
|  | Non-B | 56 | 36 (64%) | 20 (36%) | 1.25 | 0.71–2.21 | 0.439 |
| Stage at diagnosis | Chronic | 207 | 119 (57%) | 88 (43%) | 1 | - | - |
|  | Acute/early | 126 | 90 (71%) | 36 (29%) | 1.85 | 1.15–2.97 | 0.011 |
|  | Unknown | 423 | 240 (57%) | 183 (43%) | 0.97 | 0.69–1.36 | 0.858 |
| AIDS status in 2013 | Non-AIDS | 432 | 258 (60%) | 174 (40%) | 1 | - | - |
|  | AIDS | 324 | 191 (59%) | 133 (41%) | 0.97 | 0.72–1.03 | 0.831 |
| Age at diagnosis | - | - | - | - | 1.02 | 1.01–1.03 | 0.005 |
| Named partners | - | - | - | - | 0.89 | 0.82–0.96 | 0.002 |
| Genotyped partners | - | - | - | - | 0.81 | 0.60–1.10 | 0.181 |

Hetero, heterosexual; MSM, men who have sex with men; IDU, injecting drug user

^1^Demographic categories reflect index case

^2^Univariate odds ratio
