# Supplementary material for: Social and Genetic Networks of HIV-1 Transmission in New York City
Source: PLoS Pathog. 2017 Jan 9;13(1):e1006000. doi: 10.1371/journal.ppat.1006000 (PMC5221827; doi:10.1371/journal.ppat.1006000)
Supplement: S3 Table — (DOCX) [file ppat.1006000.s003.docx]

**Table S3. Multivariate regression analysis of index cases being genetically-linked to their named partners.**

| **Demographic** | **Category^1^** | **Index cases** | β | **95% confidence interval** | ***p*-value** |
| --- | --- | --- | --- | --- | --- |
| Total | - | 756 | - | - | - |
| Risk | Hetero (F) | 215 | 1 | - | - |
|  | Hetero (M) | 92 | 0.01 | -0.11 – 0.12 | 0.900 |
|  | MSM | 339 | -0.33 | -0.41 – -0.25 | <0.001 |
|  | IDU (F) | 15 | -0.22 | -0. 46 – 0.13 | 0.064 |
|  | IDU (M) | 35 | -0.35 | -0.51 – -0.19 | <0.001 |
|  | Other/Unknown | 60 | 0.03 | -0.11 – 0.16 | 0.705 |
| Race | Black | 390 | 1 | - | - |
|  | Hispanic | 306 | 0.14 | 0.06 – 0.21 | <0.001 |
|  | White/Other | 60 | 0.17 | 0.04 – 0.29 | 0.011 |
| Country of birth | USA | 489 | 1 | - | - |
|  | Foreign | 211 | 0.03 | -0.50 – 0.11 | 0.487 |
|  | US Dependency | 54 | -0.04 | -0.18 – 0.09 | 0.531 |
|  | Unknown | 2 | -0.17 | -0.80 – 0.46 | 0.605 |
| Subtype | B | 700 | 1 | - | - |
|  | Non-B | 56 | 0.00 | -0.13 – 0.12 | 0.943 |
| Stage at diagnosis | Chronic | 207 | 1 | - | - |
|  | Acute/early | 126 | 0.10 | 0.00 – 0.20 | 0.057 |
|  | Unknown | 423 | 0.0 | -0.78 – 0.75 | 0.972 |
| AIDS status in 2013 | Non-AIDS | 432 | 1 | - | - |
|  | AIDS | 324 | 0.85 | -0.10 – 0.04 | 0.355 |
| Age at diagnosis | - | - | 0.00 | 0.00 – 0.00 | 0.300 |
| Named partners | - | - | -0.14 | -0.03 – 0.00 | 0.031 |
| Genotyped partners | - | - | -0.03 | -0.11 – 0.04 | 0.377 |

Hetero, heterosexual; MSM, men who have sex with men; IDU, injecting drug user

^1^Demographic categories reflect index case
