# Supplementary material for: Social and Genetic Networks of HIV-1 Transmission in New York City
Source: PLoS Pathog. 2017 Jan 9;13(1):e1006000. doi: 10.1371/journal.ppat.1006000 (PMC5221827; doi:10.1371/journal.ppat.1006000)
Supplement: S4 Table — (DOCX) [file ppat.1006000.s004.docx]

**Table S4. Logistic regression analysis of index case being genetically-linked to at least one of their named partners for index cases who named only 1 partner.**

| **Demographic** | **Category^1^** | **Index cases** | **Named ≥ 1 genetically linked partner, n (%)** | **Did not name a genetically linked partner, n (%)** | **Odds Ratio^2^** | **95% confidence interval** | ***p*-value** |
| --- | --- | --- | --- | --- | --- | --- | --- |
| Total | - | 419 | 281 (67%) | 138 (33%) | - | - | - |
| Risk | Hetero (F) | 145 | 114 (79%) | 31 (21%) | 1 | - | - |
|  | Hetero (M) | 59 | 43 (73%) | 16 (27%) | 0.79 | 0.38–1.65 | 0.529 |
|  | MSM | 151 | 80 (53%) | 71 (47%) | 0.30 | 0.17–0.51 | <0.001 |
|  | IDU (F) | 10 | 6 (60%) | 4 (40%) | 0.44 | 0.11–1.74 | 0242 |
|  | IDU (M) | 17 | 9 (53%) | 8 (47%) | 0.29 | 0.09–0.87 | 0.027 |
|  | Other/Unknown | 37 | 29 (78%) | 8 (22%) | 1.24 | 0.50–3.05 | 0.642 |
| Race | Black | 208 | 128 (62%) | 80 (38%) | 1 | - | - |
|  | Hispanic | 182 | 130 (71%) | 52 (29%) | 1.61 | 0.97–2.68 | 0.067 |
|  | White/Other | 29 | 23 (79%) | 6 (21%) | 3.25 | 1.20–8.89 | 0.022 |
| Country of birth | USA | 259 | 163 (63%) | 96 (37%) | 1 | - | - |
|  | Foreign | 125 | 94 (70%) | 31 (30%) | 1.57 | 0.92–2.68 | 0.101 |
|  | US Dependency | 33 | 23 (70%) | 10 (30%) | 0.83 | 0.33–2.508 | 0.691 |
|  | Unknown | 2 | 1 (50%) | 1 (50%) | 0.40 | 0.02–8.13 | 0.552 |
| Subtype | B | 381 | 255 (67%) | 126 (33%) | 1 | - | - |
|  | Non-B | 38 | 26 (68%) | 12 (32%) | 0.90 | 0.42–1.93 | 0.778 |
| Stage at diagnosis | Chronic | 128 | 80 (63%) | 48 (37%) | 1 | - | - |
|  | Acute/early | 68 | 54 (79%) | 14 (21%) | 1.94 | 0.92–4.08 | 0.080 |
|  | Unknown | 223 | 147 (66%) | 76 (34%) | 1.25 | 0.76–2.05 | 0.383 |
| AIDS status in 2013 | Non-AIDS | 225 | 155 (69%) | 70 (31%) | 1 | - | - |
|  | AIDS | 194 | 126 (65%) | 68 (35%) | 0.81 | 0.51–1.28 | 0.364 |
| Age at diagnosis | - | - | - | - | 1.01 | 0.99–1.03 | 0.400 |

Hetero, heterosexual; MSM, men who have sex with men; IDU, injecting drug user

^1^Demographic categories reflect index case

^2^Model adjusted odds ratio
